# The Radiation Therapist profession through the lens of new technology: A practice development paper based on the ESTRO Radiation Therapist Workshops

**DOI:** 10.1016/j.tipsro.2024.100243

**Published:** 2024-03-15

**Authors:** Michelle Leech, Alaa Abdalqader, Sophie Alexander, Nigel Anderson, Barbara Barbosa, Dylan Callens, Victoria Chapman, Mary Coffey, Maya Cox, Ilija Curic, Jenna Dean, Elizabeth Denney, Maeve Kearney, Vincent W.S. Leung, Martina Mortsiefer, Eleftheria Nirgianaki, Justas Povilaitis, Dimitra Strikou, Kenton Thompson, Maud van den Bosch, Michael Velec, Katrina Woodford, Monica Buijs

**Affiliations:** a Applied Radiation Therapy Trinity, Discipline of Radiation Therapy, Trinity College Dublin, Ireland; b Trinity St. James’s Cancer Institute, Dublin, Ireland; c Burjeel Medical City, UAE, Abu Dhabi, United Arab Emirates; d The Royal Marsden NHS Foundation Trust and The Institute of Cancer Research, Sutton, United Kingdom; e Department of Radiation Oncology, Olivia Newton-John Cancer Wellness & Research Centre - Austin Health, Heidelberg, Australia; f Escola Internacional de Doutoramento, Universidad de Vigo, Spain; g Medical Physics, Radiobiology and Radiation Protection Group, IPO Porto Research Center (CI-IPOP), Porto Comprehensive Cancer Center (Porto.CCC) & Rise@CI-IPOP (Health Research Network), Porto, Portugal; h University Hospital Leuven, Department of Radiation Oncology, Leuven, Belgium; i KU Leuven, Laboratory of Experimental Radiotherapy, Leuven, Belgium; j Clatterbridge Cancer Centre, Liverpool, United Kingdom; k Auckland City Hospital, Auckland, New Zealand; l Radiosurgery and Stereotactic Radiotherapy Department, University Clinical Center of Serbia, Belgrade, Serbia; m Universitätsspital Zürich. ETH, Zürich, Switzerland; n Department of Health Technology and Informatics, The Hong Kong Polytechnic University, Hong Kong; o Siemens Healthineers, Forchheim, Germany; p General Anti-Cancer Hospital “Agios Savvas”, Athens, Greece; q The Hospital of Lithuanian University of Health Sciences Kauno klinikos, Kaunas, Lithuania; r Radiation Oncology Unit, University and General Attikon Hospital, Athens, Greece; s Department of Radiation Therapy Services, Peter MacCallum Cancer Centre, Melbourne, Australia; t MAASTRO Clinic, Maastricht, the Netherlands; u Princess Margaret Cancer Centre, University Health Network, Toronto, Canada; v Department of Radiation Oncology, University of Toronto, Toronto, Canada; w Department of Medical Imaging and Radiation Sciences, Monash University, Clayton, Australia; x InHolland Haarlem, University of Applied Science, Haarlem, the Netherlands

**Keywords:** Radiation therapist, Technological advances, Scope of practice

## Abstract

Technological advances in radiation therapy impact on the role and scope of practice of the radiation therapist. The European Society of Radiotherapy and Oncology (ESTRO) recently held two workshops on this topic and this position paper reflects the outcome of this workshop, which included radiation therapists from all global regions.

Workflows, quality assurance, research, IGRT and ART as well as clinical decision making are the areas of radiation therapist practice that will be highly influenced by advancing technology in the near future. This position paper captures the opportunities that this will bring to the radiation therapist profession, to the practice of radiation therapy and ultimately to patient care.

## Highlights

• This paper describes the future role of the radiation therapist in light of new technological advances, including artificial intelligence.
• The paper is co-authored by radiation therapists from all world regions, giving a unique global perspective on the future role of the profession.
• Workflows, QA, research, IGRT-ART and clinical decision making are areas of practice that will be influenced by advancing technology.

## Introduction

New technology, including Artificial Intelligence (AI) has, and will continue to, revolutionise how we practice radiation therapy (RT). The ability and capacity of AI to detect, identify, process and remember an increasing number of relevant variables has been demonstrated in many settings and linked human knowledge, skills and empathy can be of immense benefit to patient care [1], [2].

Based on a recent European Society of Radiotherapy and Oncology (ESTRO) workshop, this practice development paper will discuss the areas where new technology will progress the profession of radiation therapy. We believe that AI in particular, should be viewed as one enabling technology for radiation therapists contributing to the team effort to personalise patient treatment and care.

To ensure successful development and implementation of new technology in RT, it is essential that radiation therapists are involved at all stages of the process. Radiation therapists, who understand the intricacies of the RT workflow, are in an ideal position to identify areas where further technological intervention is most needed or will have greatest impact. Prioritising solutions that address clinical and workforce needs are vital [3].

Radiation therapists have an in-depth knowledge of the patient, clinical and technical data collected during RT, enabling them to proficiently identify, sort and export data relevant for the application of new technologies. Furthermore, radiation therapists can enhance the quality of the data collected, for example by optimising image quality, contours or dosimetry, where the performance of machine learning greatly depends on the quality of input data [3], [4].

As an end-user, radiation therapists are fundamental to the clinical application of technological solutions. They are in an opportune position to critique the usability and suitability of such tools, validate their outputs and modify protocols and workflows accordingly. The relationship between radiation therapist and patient provides opportunity to deliver training and guidance to patients on advanced technology, to ensure full understanding of their application and associated ethical considerations. By doing so, the radiation therapist takes an active role in promoting patient safety and ensuring that these tools are used effectively and responsibly in the context of RT.

Radiation therapists bear responsibility for treatment preparation, as part of the multidisciplinary team, and treatment delivery. It is therefore essential that they develop the technical skills and knowledge needed to evaluate and troubleshoot technology effectively [5]. Addressing these challenges requires education and training opportunities at undergraduate and postgraduate level to improve digital literacy and skills [6], promoting the benefits of new technology and engaging radiation therapists in the development of technological solutions to increase their acceptability and perceived effectiveness [3].

In this practice development paper, we will discuss how AI and other technological advances will move the profession of radiation therapy forward with specific reference to improvement in quality consistency and workflow, research, image guided radiation therapy (IGRT), adaptive radiation therapy (ART) and clinical decision making.

## Radiation therapy workflows

Technology has the potential to serve as a digital transformation tool [7]. One example is the replacement of repetitive and time-consuming tasks prone to interobserver variability at various stages of the RT workflow **(**Table 1**)** to increase productivity and workload efficiency [8]. This would provide opportunity for traditionally rigidly-scheduled radiation therapists time to focus on other work. This may include psychosocial patient care, RT toxicity management and research. This will support the development of advanced practitioner roles, ultimately improving practice and outcomes and enhancing the patient experience.

**Table 1:** Summary of potential applications of new technology in the radiation therapy workflow.

RT stage/process	Example
Simulation and treatment planning	Diagnostic image interpretation – radiomics Image acquisition, quality and registration Personalisation of patient setup, dose, fractionation, organ at risk (OAR) tolerances Automated target and OAR delineation Automated knowledge-based planning Prediction of dose, efficacy, toxicity and prognosis
Quality assurance	Identification of the correct patient and linking to ancillary equipment, collision potential Monitoring machine output and performance Detecting systematic and random errors
Patient information and care	Patient identification Assistance in the delivery of patient education and advice Toxicity surveillance and escalation Supporting delivery of clinical and psychosocial care to patients
Patient monitoring	Continuous monitoring of patient position, target location and physical and biological features
Radiation therapy delivery	Improved image acquisition Real-time monitoring of targets Online plan selection Online plan adaptation Online dose monitoring and accumulation

## Quality

Technology has the potential to improve the quality and consistency of RT treatments. Automated treatment plans eliminate the dependence on the skill and training of the planner/dosimetrist, consistently producing high quality plans [9]. This could improve parity across patients, departments, health networks and regions. Compiling, sorting and analysing internationally accrued RT datasets is also more conceivable with new technology such as AI, as it offers potential to leverage new information and insights, which could transform patient information and improve patient care and outcomes [8]. The radiation therapist will therefore take a higher level role in evaluation and decision making.

## Research

As experts in their field, radiation therapists are ideally suited to carry out research into their own practice and should be encouraged to participate in all research activities. Further encouragement to participate in research is needed as currently only 30–40 % of radiation therapists are involved in all aspects of the research process [10]. Research participation barriers include a lack of research skills, knowledge and time constraints [10], [11], lack of confidence in ability to identify research questions and carry out research to the level required, understaffed departments, and other role demands [2]. These challenges must be overcome so that radiation therapists develop the skills and knowledge to be involved in and meaningfully contribute to high quality research and innovation.

To facilitate this, research training programmes, ranging from undergraduate to post-doctoral levels need to be developed and supported. Increasing the currently small proportion of doctorally-educated radiation therapists should facilitate greater research leadership, radiation therapists functioning as independent principal investigators, and more meaningful collaborations with other scientists and clinicians [12], [10].

Re-addressing staffing models and increasing the use of automation to improve workflow efficiency also offers opportunity to increase radiation therapist participation in research. Increasing the involvement of radiation therapists in research and innovation can lead to advancements that directly impact service delivery and outcomes for patients positively as well as providing the potential for radiation therapist-led clinical and academic projects [13], [14]. Fostering industry partnerships may assist radiation therapists to undertake research and offer benefits such as early user input to software development to improve end user acceptance. Some funding barriers may also be navigated though industry collaboration.

Areas of research that would benefit from radiation therapist involvement include those in patient care, AI, automation, image guided and adaptive radiation therapy, person centred care and implementation science.

## Image guided radiation therapy (IGRT) and adaptive radiation therapy (ART)

Radiation therapist-led conventional IGRT is standard practice in many regions, which is rapidly moving towards more complex scenarios such as stereotactic ablative radiation therapy (SABR) and adaptive radiation therapy (ART)[15], [16]. The shifting radiation therapist role within IGRT/ART is underpinned by successful training and credentialing programs, where radiation therapists have taken on the responsibility for technical decision making. Precedence has been established initially for plan-of-the-day ART and now increasingly online ART [17], [18], [19], [20]. Autonomous radiation therapists have enabled much needed multi-centre clinical trials and enhanced radiation therapist decision making in IGRT and ART [21].

A common challenge for radiation therapists moving forward in ART is that the responsibility for clinical decision making is often legally with the radiation or clinical oncologist (RO/CO). Decisions are based on a variety of factors (e.g., treatment intent, dosimetric criteria, treatment timing, and patient performance status) [22], [23], [24]. Often radiation oncologists focus on knowledge transfer and decision support between themselves without inclusion of the radiation therapist [25], [26], [27]. For groups moving to online ART, having RO presence at the linear accelerator is unsustainable, and on-call RO support is ineffective. A strong functioning team is essential for continuity of care, team and patient communication skills with a radiation therapist led service playing a key role [28], [29].

Given the shift towards online ART, it is envisioned radiation therapists will work with even greater autonomy. The radiation therapist role may evolve to be more like that of other health and social care professions who are members of the care team but are responsible for filling the prescribed treatment, while the RO oversees and monitors its clinical outcome. To achieve this, the radiation therapist will need to move beyond a task shifting role, to a more consultive role, bringing what is currently considered as advanced practice functioning to the standard practice radiation therapy team. There are many examples of advanced practice radiation therapists demonstrating a high level of autonomy and expert skill in formulating clinical decisions and appropriate patient management [30], [31], [32], [33]. They provide comprehensive supportive care and toxicity management, streamline complex processes, reduce handovers and team interruptions, and improve continuity of care. To further enable this support it would be ideal if the radiation therapist in ART joined the multidisciplinary team that conducted the new patient consultation [34], [35].

Given the increasing complexity of IGRT/ART, the radiation therapist could perform an RT-consultation after the RO/CO medical consultation. Here patient-specific modality and strategies would be decided upon in collaboration with the care team (e.g., photon vs proton, CT or MR-guidance and motion management). There is an element of stewardship of resources as well, where radiation therapists would aid in the evidence based application of the most appropriate technology. Radiation therapists can improve patient understanding of technical processes, reducing high patient concerns with coordination of care by adapting education materials, and communication styles to patient-specific needs [36], [37], [38]. Patients require skilled and confident radiation therapists, with whom they are familiar, in order to manage anxiety with complex treatments and unexpected situations. This is expected to become even more important with intensive IGRT/ART strategies [39].

We envisage that radiation therapists may have a role in driving adaptation based on clinical outcomes from patient reported outcomes (PROs), clinical response, functional imaging and new fractionations (e.g. PULSAR). ART will likely increasingly rely on biological changes over anatomic changes through the use of functional imaging [40]. Functional changes can occur earlier than anatomic changes providing additional opportunity to intervene earlier in the treatment trajectory. For this reason, increasing adoption of multimodal imaging such as MRI is likely with frequent monitoring of volume, shape and biological characteristics and regularly adapting the treatment plan based on the observed treatment response [16]. Radiation therapist education that includes increasing knowledge and application of multimodal imaging and therapy, and advanced treatment planning will enable increasing radiation therapist assessment and analysis of clinical and radiological outcomes.

A task shifting is also expected downstream from radiation therapists to AI to streamline workflow and enable adaptive strategies that are otherwise resource intensive. AI based automation will greatly expand the application of ART by further helping to reduce treatment times and further reduce the burden on RO/COs. All radiation therapists will require AI literacy to explain the application of AI in IGRT/ART to the patient in plain language and reassure patients on its safety and quality. Radiation therapists practice at the patient/technology interface and should not be removed from AI processes as they will have a role in their oversight and intervention [41], [42].

Radiation therapists, as end-users of IGRT and ART technologies, need to be meaningfully engaged early in the research and development (R&D) process. There are numerous opportunities for radiation therapists to lead and contribute to IGRT/ART R&D. IGRT is widely adopted by radiation therapists, and naturally this can be extended to multi-modal imaging and therapy, AI-assisted workflows, radiation therapist training and education and patient outcomes.

## Clinical decision making

Clinical decision making is:

“a balance of known best practice (the evidence, the research), awareness of the current situation and environment, and knowledge of the patient. It is about 'joining the dots' to make an informed decision” [43].

Clinical decision making is a consistent presence along the radiation therapy pathway, with treatment approaches being evidence based and the provision of appropriate patient care reliant on radiation therapist awareness of the current situation for that patient at that point in the treatment pathway.

With increased implementation of innovative technology into the RT pathway, the question as to who will make the clinical decisions is frequently posed. We believe that this is an opportunity for radiation therapists to embrace new roles through the lens of innovative technology with routine tasks shifting to AI based systems, all the time under the control and judgement of the radiation therapist. They are also ideally positioned to lead technology implementation and use, safely and effectively in clinical practice.

With radiation oncology referrals increasing exponentially since the Covid-19 pandemic has ended and the increased use of hypofractionated regimes, efficiency in clinical decision making in a busy fast paced clinical environment is key [44], [45]. Innovative technology can become another part of the established multidisciplinary team. Radiation therapists already have the technical expertise to make confident clinical decisions in the treatment they deliver as AI based contouring, plan optimisation, gating and auto-matching systems are already in routine practice. A clear infrastructure will support radiation therapists in their decisions within the boundary of standardised practice, but autonomous clinical decision making will be essential.

To maximise the implementation of new technologies and techniques on the linear accelerator, pre-treatment preparation such as positioning, immobilisation and scan acquisitions must be robust and optimal. New technology means advances in CT acquisition and the increased implementation of MRI and PET-CT imaging, as well as functional imaging. Radiation therapists must have sufficient clinical decision making skills to decide which scans are indicated for each treatment site, being cognisant of the planning aims for a specific patient. The use of post-processing functions also provide opportunities to create additional datasets for treatment planning which may benefit the planning process.

Within the multidisciplinary team, radiation therapists are in the unique position of having daily contact with their patients. As a constant part of the treatment journey, radiation therapists build trusting relationships with patients and can be considered the ‘gatekeeper of patient care’, in the exclusive position to identify changes in behaviour, clinical status and general wellbeing of patients [46]. Patient management is already within the current radiation therapist scope of practice [47] with referral pathways for psychosocial or specialised interventions available when required. Innovation in radiation oncology information systems and electronic medical records provide up-to-date patient records while apps and wearables have been shown to empower patients to participate in their own care [48].

In an environment which increasingly embraces technology, the overall care of the patient must still be the focus. Technology (AI included) should be regarded as another member of the team facilitating increased face-to-face interaction between radiation therapists and patients.

## Conclusion

This practice development paper, authored by radiation therapists globally, puts forward an international consensus on the likely progression of our profession in the context of new and emerging technologies.

Radiation therapists are well equipped to appreciate the potential and limitations of new technologies from a practical perspective and are ideally positioned to lead implementation and use technology safely and effectively in clinical practice. Competencies related to the technical application of radiotherapy, alongside patient evaluation and care, must be embedded in radiation therapists core knowledge and skills and should form the basis of all radiation therapist education programmes.

## Declaration of competing interest

The authors declare the following financial interests/personal relationships which may be considered as potential competing interests: The lead author is EIC of tipsRO. The following authors are editorial board members of tipsRO: Nigel Anderson, Mary Coffey, Kenton Thompson, Michael Velec and Monica Buijs. Given that this manuscript is reporting on an ESTRO activity, the associated paper is for publication in an ESTRO journal. As the activity is specific to radiation therapists, tipsRO is the most appropriate journal for this report.
